# Prophylactic relationship between mental health disorder symptoms and physical activity of Royal Canadian Mounted Police Cadets during the cadet training program

**DOI:** 10.3389/fpsyg.2023.1145184

**Published:** 2023-05-16

**Authors:** Taylor Teckchandani, Rachel L. Krakauer, Katie L. Andrews, J. Patrick Neary, Jolan Nisbet, Robyn E. Shields, Kirby Q. Maguire, Laleh Jamshidi, Tracie O. Afifi, Lisa M. Lix, Shannon Sauer-Zavala, Gordon J. G. Asmundson, Gregory P. Krätzig, R. Nicholas Carleton

**Affiliations:** ^1^Canadian Institute for Public Safety Research and Treatment-Institut Canadien de recherche et de traitement en sécurité publique (CIPSRT-ICRTSP), University of Regina/Université de Regina, Regina, SK, Canada; ^2^Anxiety and Illness Behaviours Lab, Department of Psychology, University of Regina, Regina, SK, Canada; ^3^Faculty of Kinesiology and Health Studies, University of Regina, Regina, SK, Canada; ^4^Department of Community Health Sciences, University of Manitoba, Winnipeg, MB, Canada; ^5^Department of Psychology, University of Kentucky, Lexington, KY, United States; ^6^Department of Psychology, University of Regina, Regina, SK, Canada

**Keywords:** posttraumatic stress injuries, exercise, protective factors, Royal Canadian Mounted Police, apple watch

## Abstract

**Objective:**

Royal Canadian Mounted Police report experiencing extremely frequent potentially psychologically traumatic events (PPTE). In a recent study, approximately half of participating RCMP screened positive for one or more mental disorders, which is approximately five times the diagnostic proportion for the general Canadian population. Increased reporting of mental health symptoms been linked to PPTE exposures. Programs promoting physical activity may be useful interventions to supplement or pair with mental health interventions, providing anxiolytic, antidepressant, and stress-buffering effects. The current study was designed to assess the relationship between physical activity behaviors and reported mental health disorder symptoms of cadets during the Royal Mounted Canadian Police (RCMP) Cadet Training Program (CTP). The current study also examined the relationship between exercise and mental health disorder symptoms of cadets during the CTP.

**Methods:**

The study included data from 394 cadets (76.1% male). An analysis of variance (ANOVA) and a series of *t*-tests were used to assess several differences across sociodemographic groups. Bivariate Spearman’s Rank correlations were performed between the average number of active calories burned per day, as recorded by Apple Watches, and changes in self-reported mental health disorder symptoms (i.e., Generalized Anxiety Disorder [GAD], Major Depressive Disorder [MDD], Posttraumatic Stress Disorder [PTSD], Social Anxiety Disorder [SAD]. Alcohol Use Disorders [AUD], Panic Disorder [PD]) from pre-training (starting the CTP) to pre-deployment (completing the CTP) 26 weeks later.

**Results:**

There were statistically significant correlations between physical activity and self-reported mental health disorder symptom scores during CTP. Cadets who performed more physical activity from pre-training to pre-deployment had statistically significantly greater decreases in symptoms of GAD (*ρ* = −0.472, *p* < 0.001), MDD (*ρ* = −0.307, *p* < 0.001), PTSD (*ρ* = −0.343, *p* < 0.001), and AUD (*ρ* = −0.085, *p* < 0.05). There was no statistically significant relationship between physical activity and changes in PD symptoms (*ρ* = −0.037, *p* > 0.05). There were also no statistically significant relationships between pre-CTP mental health disorder symptom scores and the volume of physical activity performed during CTP.

**Conclusion:**

There was evidence of a significant relationship between reductions in mental health disorder symptom scores and physical activity during the 26-week CTP. The results highlight the role that exercise can play as an important tool for reducing mental health disorder symptoms, considering there was no relationship between pre-CTP baseline mental health scores and physical activity performed during CTP. Further research is needed to understand differences in physical activity behaviours among cadets and serving RCMP.

## 1. Introduction

Public safety personnel (PSP), such as firefighters, paramedics, municipal police, and the Royal Canadian Mounted Police (RCMP), work to ensure the safety of the public in Canada (Canadian Institute for Public Safety Research and Treatment (CIPSRT), 2019). PSP frequently experience potentially psychologically traumatic events (PPTE) as a result of their professional responsibilities (Carleton et al., 2019). PPTEs involve either direct or indirect exposure to serious injury, sexual assault, or actual or imminent death (Canadian Institute for Public Safety Research and Treatment (CIPSRT), 2019). Higher prevalence of mental health disorder symptoms, such as posttraumatic stress disorder (PTSD) and generalized anxiety disorder (GAD), has frequently been reported to be associated to PPTE exposures in PSP (Carleton et al., 2019). RCMP Officers have previously reported high prevalence of exposures to PPTEs (Carleton et al., 2019), with 23% screening positive for GAD, a rate three times that of the general Canadian population (8.7%) (Pelletier et al., 2017).

Longitudinal (Carleton et al., 2018) and cross-sectional (Leppin et al., 2014; Robertson et al., 2015; Carleton et al., 2020) research results suggest current programs to support PSP mental health typically concentrate on raising awareness, reducing stigma, and encouraging help-seeking behaviors (Papazoglou and Andersen, 2014); however, the programs seem to produce effects that are small to moderate, time limited, often not statistically significant, and variable based on the many individual characteristics and program delivery fidelity (Carleton et al., 2018; Fikretoglu et al., 2019; Szeto et al., 2019). There is also limited data supporting the usefulness of the several interventions suggested to promote PSP mental health, in part because control conditions have been absent from evaluations of effectiveness (Zylberberg et al., 2011; Anderson et al., 2020; Di Nota et al., 2021). The interventions are also often focused on increasing individual PSP knowledge or promoting individual help-seeking, rather than providing specific and tailored skills (Anderson et al., 2020). Most of the current intervention programs also do not outline or measure changes in the individual differences being targeted (e.g., symptoms; biopsychosocial risk and resiliency factors), the methods being used to change those individual differences, or the mechanisms of action underlying the intervention (Anderson et al., 2020; Wild et al., 2020).

Physical activity has been reported to moderately decrease symptoms of mood-, anxiety- (Grasdalsmoen et al., 2020), and trauma-related (Rosenbaum et al., 2015; Whitworth and Ciccolo, 2016; Whitworth et al., 2019) disorders, and minimize symptoms of stress, panic, and negative affect (Asmundson and Katz, 2009; Fetzner and Asmundson, 2014; Powers et al., 2015; Abdollahi et al., 2017; Mason et al., 2019). Physical activity has anxiolytic effects due to repeated exposure to anxiety-related somatic sensations (Stubbs et al., 2017a,b). Increased physical activity can also decrease symptoms of hyper-arousal related to PTSD (Vancampfort et al., 2016a,b). Based on the current evidence, programs promoting physical activity may be a useful, accessible, and acceptable intervention or adjuvant for mental health training, providing PSP with anxiolytic, antidepressant, and stress-buffering effects.

Despite the substantial body of literature available, there is limited research evaluating the role of physical activity on symptoms of mental health disorders among police and other PSP. There is very limited evidence regarding physical activity and mental health of police recruits starting their careers. Assessing the role of physical activity may be particularly critical for police officers who engage in high levels of unstructured movement as part of their occupational duties. Results from examining physical activity among police have been mixed, with some studies reporting associations between higher physical activity and lower psychological stress (Craun et al., 2014; Ramey et al., 2014), and other studies finding no associations (Meckes et al., 2021).

The current study examined the relationship between levels of physical activity and changes in self-report mental health disorder symptoms from the start of the RCMP Cadet Training Program (CTP) to graduation from the program 26 weeks later. Based on previous research, physical activity was expected to be inversely associated with changes in mental health disorder symptoms of mood-, anxiety-, and trauma-related disorders.

## 2. Materials and methods

### 2.1. Procedure

The RCMP Study (Carleton et al., 2022) provides an opportunity to address several gaps regarding physical activity and mental health among a sample of cadets completing the CTP. The RCMP Study (Carleton et al., 2022) is a multi-modal mental health solution that incorporates evidence-based biopsychosocial evaluations and evidence-based integrated cadet mental health training. Participating cadets completed self-report and biometric assessments. The biometric assessments provided a relative assessment of health based on data collected from Apple Watches provided to participants. The collected data allow for assessing associations between individual differences in psychological and physiological variables, such as physical activity and mental health disorder symptoms. Full details on the RCMP Study can be found in the associated protocol paper (Carleton et al., 2022). The RCMP Study was approved by the University of Regina Institutional Research Ethics Board (file No. 2019–055) and the RCMP Research Ethics Board (file No. SKM_C30818021312580). The RCMP Study was also approved through a Privacy Impact Assessment as part of the overall approval National Administration Records Management System (NARMS) (file No. 201611123286) and Public Services and Procurement Canada (PSPC) (file No. 201701491/M7594174191).

### 2.2. Data and sample

Participants were RCMP cadets (*n* = 394; 76.1% male) who completed the 26-week CTP. The current paper focuses on cadets who completed the CTP and provided Apple Watch data as part of the larger RCMP Study. To qualify for the CTP, cadets must be between the ages of 19 and 57 years, Canadian citizens or permanent residents, and fluent in either English or French (Royal Canadian Mounted Police, 2022). In addition, cadets must pass screening procedures, such as security checks, physical exams, polygraph tests, and minimum physical requirements. There were no restrictions requiring those who were otherwise eligible for the CTP to be excluded.

### 2.3. Self-report measures

Mental health disorder symptoms were assessed using web-delivery of the Full Assessment at pre-training and pre-deployment (see protocol paper for design details; (Carleton et al., 2022), which included the PTSD Check List 5 [PCL-5 (Weathers et al., 2013)]; the 9-item Patient Health Questionnaire [PHQ-9 (Kroenke et al., 2001)]; the Panic Disorders Symptoms Severity Scale, Self-Report [PDSS-SR (Shear et al., 1997)]; the 7-item Generalized Anxiety Disorder scale [GAD-7 (Spitzer et al., 2006)]; and the Alcohol Use Disorders Identification Test [AUDIT (Saunders et al., 1993)]. Participant reported symptoms per instructions for each scale: PHQ-9 symptoms were reported for the previous 14 days, PDSS-SR symptoms for the previous 7 days, GAD-7 symptoms for the previous 14 days, and AUDIT symptoms for the last year. Pre-CTP baseline mental health symptom scores were calculated along with within participant change scores for each of the PCL-5, PHQ-9, PDSS-SR, GAD-7, and AUDIT by subtracting the pre-deployment (i.e., Time 2) scores from the pre-training (i.e., Time 1) scores. Pre-training and post-training mental health symptom scores for this sample can be found in the associated mental health monitoring paper (Shields et al., 2023).

### 2.4. Sociodemographic variables

Sociodemographic characteristics, including sex (i.e., male and female), age (i.e., 19 to 29 years, 30 to 39 years, 40 to 49 years, and 50 to 59 years), marital status (i.e., single, separated/divorced, and married/common-law), province of residence [i.e., Western Canada (British Columbia, Alberta, Saskatchewan, Manitoba), Eastern Canada (Ontario, Quebec), Atlantic Canada (Newfoundland & Labrador, Prince Edward Island, Nova Scotia, New Brunswick), or Northern Territories (Yukon, Northwest Territories, Nunavut)], and education (i.e., high school graduate or less, some post-secondary school, and university degree/4-year college or higher) were used for detailed descriptions of groupwise comparisons and covariates (Carleton et al., 2022).

### 2.5. Physical activity measures

Cadets were equipped with Apple Watch devices (series 4 and series 5) to observe biophysiological measures (i.e., heart rate, heart rate variability, step count, sleep patterns) and physical activity measured by active calories burned, supported by a dedicated Apple iPhone. Cadets used the iPhones with wi-fi at no cost, and could use the iPhones as personal phones, but were not provided voice or data plans. Apple Watch recordings were downloaded via an encrypted process to the secured University of Regina server for off-line processing and analyses.

The explicit choice to use active calories burned as a single marker of physical activity is based on the existing literature highlighting the reliability of active calories burned when observed within participants in a longitudinal framework (Dooley et al., 2017; Shcherbina et al., 2017; Bai et al., 2018, 2021; Murakami et al., 2019; Düking et al., 2020). The Apple Watch’s proprietary algorithm produced caloric expenditure estimates in both clinical and free-living environments that appeared superior to those of six other commercial devices (Shcherbina et al., 2017). The Apple Watch achieved the lowest overall error in both estimates of heart rate and caloric expenditure at rest, low intensity, and moderate to vigorous physical activity, but all wearable devices appear inferior to gold standard methods, such as the metabolic chamber and doubly labeled water methods (Dooley et al., 2017; Shcherbina et al., 2017; Bai et al., 2018, 2021; Murakami et al., 2019; Düking et al., 2020). Despite the degree of error from gold standard methods of analyzing caloric expenditure, said error is stable within participants and thus provides the basis for a trend analysis. Accelerometer based metrics such as step count or estimated flights climbed have often been reported with greater within and between participant error than active calories burned (Dooley et al., 2017; Bai et al., 2018, 2021; Düking et al., 2020).

### 2.6. Statistical analyses

Sociodemographic characteristics for study participants, such as sex, age, marital status, province of residence, and education, were included for descriptive statistics during pre-training and comparisons between sociodemographic groups. An analysis of variance (ANOVA) and a series of *t*-tests were used to check for differences across sociodemographic groups. Holm-Bonferroni adjustments were made to alpha values to take into consideration the familywise error rate in multiple comparisons from *post hoc* testing. Cohen’s *d* values are presented as standardized effect sizes for *t*-tests to display the standardized differences between two means, interpreting effect sizes as small (*d* = 0.20), medium (*d* = 0.50), and large (*d* = 0.80) (Cohen, 2013). After taking into account the variance explained by other factors in the model, partial eta squared ($\eta_{p}^{2}$) is presented for interpreting ANOVA test effect sizes as small ($\eta_{p}^{2}$=.01), medium ($\eta_{p}^{2}$=0.06), and large ($\eta_{p}^{2}$=0.14) (Cohen, 2013). Spearman’s Rank correlations were performed between pre-CTP baseline scores as well as change scores for the GAD-7, PHQ-9, AUDIT, PDSS-SR, and PCL-5, and the Apple Health variable of Active Calories Burned during CTP. Spearman’s rank correlation was chosen due to the non-normally distributed data and the observably monotonic relationship between the variables.

## 3. Results

Results of ANOVA groupwise comparisons and details of self-reported participant sociodemographics and self-reported symptom change scores are provided in Table 1. Participants were mostly male (76.1%), between the age of 19 to 29 years (59.8%), and single (47.7%) or married/common-law (50.5%). Participants were mainly from Western Canada (77.9%; i.e., British Columbia, Alberta, Saskatchewan, Manitoba) and reported having either some post-secondary school (48.5%) or a university degree, 4-year College or higher level of education (44.6%).

**Table 1:** Sociodemographic characteristics and comparison of mental health symptom screening change scores among different categories.

	%(n)^1^	AUDIT	PCL5	PDSS	GAD7	PHQ9
		Mean (SD)	Mean (SD)	Mean (SD)	Mean (SD)	Mean (SD)
Sex						
Male	76.1 (300)	−2.21 (2.54)	−4.23 (8.27)	−0.18 (1.10)	−3.22 (4.00)	−2.41 (3.31)
Female	23.9 (94)	−2.34 (2.69)	−6.03 (12.32)	−0.76 (2.28)	−3.51 (4.82)	−2.26 (4.43)
Effect size (Cohen’s *d*)		0.001	0.006	0.017**	0.001	0.000
Age						
19–29	56.3 (222)	−2.40 (2.71)	−4.46 (9.31)	−0.31 (1.45)	−3.17 (4.24)	−2.34 (3.87)
30–39	35.0(138)	−1.89 (2.34)	−5.19 (9.99)	−0.42 (1.74)	−3.64 (4.32)	−2.57 (3.41)
40–49	7.9(31)	−2.54 (2.26)	−4.62 (8.71)	^	−3.00 (3.57)	−1.67 (1.76)
50–59	^	−2.50 (3.53)	^	^	−1.00 (1.41)	−1.50 (0.71)
Effect size ($\eta_{p}^{2}$)		0.008	0.002	0.003	0.004	0.003
Marital status						
Single	47.7 (188)	−2.46 (2.85)	−5.53 (10.24)	−0.40 (1.56)	−3.44 (4.01)	−2.57 (3.96)
Separated/divorced	1.8 (7)	−3.00 (3.58)	−7.37 (9.30)	^	−6.37 (4.8)	−2.87 (2.17)
Married/common-law	50.5 (199)	−2.01 (2.21)	−3.73 (8.59)	−0.26(1.48)	−3.02 (4.34)	−2.14 (3.29)
Widowed	^	^	^	^	^	^
Effect Size ($\eta_{p}^{2}$)		0.009	0.013	0.004	0.015	0.006
Province of residence						
Western Canada (BC, AB, SK, MB)	77.9 (307)	−2.24 (2.46)	−5.47 (10.24)	−0.42 (1.72)	−3.67 (4.53)	−2.69 (3.64)
Eastern Canada (ON, QC)	16.5 (65)	−2.18 (2.71)	−3.34 (8.57)	−0.21 (1.05)	−2.78 (3.95)	−1.89 (3.83)
Atlantic Canada (PEI, NS, NB, NFL)	5.1 (20)	−2.50 (2.88)	−5.00 (7.62)	−0.26 (1.75)	−3.39 (3.44)	−2.52 (2.67)
Northern Territories (YK, NWT, NVT)	^	−2.00 (1.23)	−6.20 (11.15)	^	−2.69 (1.51)	−1.00 (0.70)
Effect size ($\eta_{p}^{2}$)		0.003	0.009	0.001	0.012	0.009
Education						
High school graduate or less	6.9 (27)	−2.20 (2.25)	−4.62 (8.81)	−0.64 (2.11)	−3.62 (4.31)	−2.68 (2.91)
Some post-secondary school	48.5 (191)	−2.45 (2.88)	−5.20 (9.32)	−0.11 (1.08)	−3.74 (4.38)	−2.61 (3.70)
University degree/4-year college or higher	44.6 (176)	−2.04 (2.32)	−3.73 (9.39)	−0.42 (1.59)	−2.65 (3.94)	−1.97 (3.72)
Effect size ($\eta_{p}^{2}$)		0.004	0.010	0.010	0.014	0.008
Total sample						

Alcohol Use Disorder Identification Test = AUDIT; Posttraumatic Stress Disorder Checklist = PCL5; Panic Disorder Symptom Scale = PDSS; Generalized Anxiety Disorder 7 = GAD7; Patient Health Questionnaire = PHQ9.

1 Total percentages may not sum to 100 and ns may not sum to 394 due to non-response or responding “other.”

*p ≤ 0.05; **p ≤ 0.01; ***p ≤ 0.001.

M (SD) represents Mean (Standard Deviation).

$\eta_{p}^{2}$represents partial Eta Square.

Lettered superscripts within each column category indicate significant differences between category groups on respective screening measure at *p* ≤ 0.05. Means followed by a common letter are not significantly different.

^Sample size between 1 and 5, so data are not presented to protect participant anonymity.

Analyses indicated statistically significant inverse relationships between self-reported mental health disorder symptom scores and the average number of active calories burned per day during CTP (Table 2). Cadets who burned more active calories per day on average during CTP had greater decreases in their GAD-7 score from pre-CTP to pre-deployment at the end of CTP (*ρ* = −0.472, *p* < 0.001), greater decreases in their PHQ-9 scores (*ρ* = −0.307, *p* < 0.001), greater decreases in their PCL-5 scores (*ρ* = −0.343, *p* < 0.001), and greater decreases in their AUDIT scores (*ρ* = −0.085, *p* < 0.05). There was no statistically significant relationship between PDSS-SR symptom scores and physical activity (*ρ* = −0.037, *p* > 0.05). There was no statistically significant relationship between pre-CTP baseline scores and the number of active calories burned per day (Table 2).

**Table 2:** Correlation coefficient comparison of mental health symptom screening change scores and active calories burned.

	%(*n*)^1^	AUDIT	PCL5	PDSS	GAD7	PHQ9
		(ρ)	(ρ)	(ρ)	(ρ)	(ρ)
Total sample
Pre-CTP score	100 (394)	0.069	−0.053	−0.118	−0.098	−0.048
Change score	100(394)	−0.085*	−0.343***	−0.037	−0.472***	−0.307***

Alcohol Use Disorder Identification Test = AUDIT; Posttraumatic Stress Disorder Checklist = PCL5; Panic Disorder Symptom Scale = PDSS; Generalized Anxiety Disorder 7 = GAD7; Patient Health Questionnaire = PHQ9.

1 Total percentages may not sum to 100 and ns may not sum to 394 due to non-response or responding “other.”

*p ≤ 0.05; **p ≤ 0.01; ***p ≤ 0.001.

M (SD) represents Mean (Standard Deviation).

(ρ) represents Spearman’s Rho.

Lettered superscripts within each column category indicate significant differences between category groups on respective screening measure at *p* ≤ 0.05. Means followed by a common letter are not significantly different.

## 4. Discussion

The current study examined associations between levels of physical activity and self-reported mental health disorder symptoms of RCMP cadets completing the CTP. The current study provides novel results regarding physical activity and mental health among cadets, which may be relevant for other PSP, especially when considering there was no relationship between physical activity performed during CTP and baseline mental health symptom scores. Demonstrably associating physical activity and mental health for cadets may support retention during the CTP and subsequent individual RCMP career resilience with physical activity as a readily accessible intervention. The current results evidence programs promoting physical activity as potentially useful, accessible, and acceptable interventions or adjuvants for cadets, RCMP officers, and other PSP to supplement other beneficial mental health activities with anxiolytic, antidepressant, and stress-buffering effects.

Consistent with extant literature, the current study highlighted the inverse relationship between self-reported mental health symptom change scores and the average quantity of active calories burned per day as measured by Apple Watches during CTP. There was a statistically significant inverse relationship between active calories burned per day and generalized anxiety disorder symptom scores (*p* < 0.001). There was no relationship between pre-CTP baseline mental health scores and the subsequent physical activity performed; accordingly, the results highlight the anxiolytic component of physical activity irrespective of activity type or clinical screening category (Wipfli et al., 2008, 2011; Wegner et al., 2014).

The second strongest relationship was between self-reported posttraumatic stress disorder symptom change scores and the average number of active calories burned per day (*p* < 0.001), highlighting the anxiolytic and potential distraction mechanisms that underpin proposed mechanisms in the extant literature (Fetzner and Asmundson, 2014; Powers et al., 2015). The third strongest relationship was between self-reported major depressive disorder symptom change scores and the average number of active calories burned (*p* < 0.001), highlighting the potential SSRI-like relationship with exercise described in the extant literature (Larun et al., 2006; Wipfli et al., 2011; Wegner et al., 2014). There was also a statistically significant relationship between self-reported alcohol use disorder change scores and the average number of active calories burned per day (*p* < 0.05), highlighting another the potentially therapeutic effect of exercise (Paffenbarger et al., 1994; Paluska and Schwenk, 2000; Wipfli et al., 2008; Mason et al., 2019).

### 4.1. Strengths and limitations

The current paper strengths include: (1) a longitudinal design in the CTP environment which promotes consistency and transparency in participation, facilitating reliable serial data collection; (2) a relatively large sample size; (3) multimodal data collection including self-report and physiological monitoring; and (4) the exceptional non-invasive ambulatory monitoring capability of Apple Watches. The current paper also has several limitations including: (1) voluntary participation creating an unknowable influence from self-selection biases; (2) symptom measurement tools that use different durations for symptom reporting periods; (3) uncommonly detailed assessments and large datasets that may increase Type I error risks from spurious correlations; (4) potential for socially desirable responding; and (5) the particularly good mental health of cadets starting the CTP (Carleton et al., 2023) may mean the current results under-represent the potential benefits of exercise for mental health.

### 4.2. Future directions

Future research should assess dose–response minimums and diminishing returns for exercise among cadets and serving RCMP. There may be important interactions between exercise and PPTE exposures among serving RCMP that can inform the potential utility of exercise as an early intervention or mitigation strategy for PTSI. Researchers could also examine interactions between exercise and numerous other variables (e.g., sleep, mental health training, mental health treatment, other biometric variables) to help nuance the application of exercise. There may also be important differences between or interactions with exercise activity types (e.g., resistance training, cardio) and mental health that necessitate additional time-series and fine-grained assessment of physical activity data made possible by ongoing data collection as part of the larger RCMP study. Survival analyses based on mental health screening variables may help to examine attrition from CTP and from the RCMP to assess the impact of exercise on vocational retention and provide guidelines for early interventions to support member health.

## 5. Conclusion

The RCMP has made the implementation of evidence-based solutions a priority to reduce the mental health challenges experienced by members (Public Safety Canada, 2019). Very little evidence exists to support the effectiveness of various interventions proposed to improve PSP mental health (Zylberberg et al., 2011; Anderson et al., 2020; Di Nota et al., 2021). The current study is the first longitudinal investigation of the relationship between physical activity and mental health symptoms for RCMP cadets in training. The results indicate that cadets who recorded more physical activity during CTP also self-reported greater reductions in mental health symptoms from pre-training to pre-deployment. The evidence highlights the need to research whether continued engagement in physical activity mitigates impacts from occupational stressors, including but not limited to PPTE, during RCMP careers. Additional groupwise and longitudinal analyses may provide unique insights about the relationships between mental health symptoms and physical activity, which may further evidence exercise as a tool for maintaining and improving mental health during the CTP and thereafter during service. Further data collection is required to understand the differences in physical activity behaviors among cadets and serving RCMP. In the interim, exercise appears to be an accessible, acceptable, and effective tool for supporting cadet mental health.

## Data availability statement

The datasets presented in this article are not readily available because data access will not be provided due to the sensitive nature of the content. Requests to access the datasets should be directed to nick.carleton@uregina.ca.

## Ethics statement

The RCMP Study was approved by the University of Regina Institutional Research Ethics Board (file No. 2019–055) and the RCMP Research Ethics Board (file No. SKM_C30818021312580). The RCMP Study was also approved through a Privacy Impact Assessment as part of the overall approval National Administration Records Management System (NARMS) (file No. 201611123286) and Public Services and Procurement Canada (PSPC) (file No. 201701491/M7594174191). The patients/participants provided their written informed consent to participate in this study.

## Author contributions

RNC, TT, JPN, RK, and KA: conceptualization. RNC, TT, RK, JPN, GA, GK, and LL: methodology. RNC, TT, RK, GK, JPN, and KA: validation. RNC, TT, RK, and JPN: formal analysis. RNC, GK, JPN, and SS-Z: investigation. RNC, GK, and SS-Z: resources. RNC, TT, GK, JPN, RK, LJ, and KM: data curation. TT, RK, RNC, KA, and JN: writing – original draft preparation. TT, RK, KA, JPN, JN, RS, KM, LJ, TA, LL, SS-Z, GA, GK, and RNC: writing – review and editing. All authors viewed and approved the submitted version of the manuscript and made substantial contributions consistent with the International Committee of Medical Journal Editors.

## Funding

The RCMP Study is funded by support from the RCMP, the Government of Canada, and the Ministry of Public Safety and Emergency Preparedness. LL is supported by a Tier I Canada Research Chair in Methods for Electronic Health Data Quality. TA is supported by a Tier I Canada Research Chair in Childhood Adversity and Resilience. The development, analyses, and distribution of the current article was made possible by a generous and much-appreciated grant from the Medavie Foundation.

## Conflict of interest

The authors declare that the research was conducted in the absence of any commercial or financial relationships that could be construed as a potential conflict of interest.

## Publisher’s note

All claims expressed in this article are solely those of the authors and do not necessarily represent those of their affiliated organizations, or those of the publisher, the editors and the reviewers. Any product that may be evaluated in this article, or claim that may be made by its manufacturer, is not guaranteed or endorsed by the publisher.
